# Investigating Exchange Bias and Coercivity in Fe_3_O_4_–γ-Fe_2_O_3_ Core–Shell Nanoparticles of Fixed Core Diameter and Variable Shell Thicknesses

**DOI:** 10.3390/nano7120415

**Published:** 2017-11-26

**Authors:** Ihab M. Obaidat, Chiranjib Nayek, Kaustuv Manna, Gourab Bhattacharjee, Imaddin A. Al-Omari, Abbasher Gismelseed

**Affiliations:** 1 Department of Physics, United Arab Emirates University, Al-Ain 15551, UAE; chiranjibnayek@gmail.com; 2 Max-Planck-Institute for Chemical Physics of Solids, Nöthnitzer Straße-40, 01187 Dresden, Germany; kaustuvmanna@gmail.com; 3 Surface Physics and Material Science Division, Saha Institute of Nuclear Physics, HBNI, 1/AF Bidhannagar, Kolkata 700064, India; gourab205@gmail.com; 4 Department of Physics, Sultan Qaboos University, P.O. Box 36, Muscat PC 123, Sultanate of Oman; ialomari@squ.edu.om (I.A.A.-O.); abbasher@squ.edu.om (A.G.)

**Keywords:** nanoparticles, shell thickness, field cooling, exchange bias, coercivity

## Abstract

We have carried out extensive measurements on novel Fe_3_O_4_–γ-Fe_2_O_3_ core–shell nanoparticles of nearly similar core diameter (8 nm) and of various shell thicknesses of 1 nm (sample S1), 3 nm (sample S2), and 5 nm (sample S3). The structure and morphology of the samples were studied using X-ray diffraction (XRD), transmission electron microscopy (TEM), and selected area electron diffraction (SAED). The direct current (DC) magnetic measurements were carried out using a superconducting quantum interference device (SQUID). Exchange bias and coercivity were investigated at several temperatures where the applied field was varied between 3 and −3 T. Several key results are obtained, such as: (a) the complete absence of exchange bias effect in sample S3; (b) the occurrence of nonconventional exchange bias effect in samples S2 and S1; (c) the sign-change of exchange bias field in sample S2; (d) the monotonic increase of coercivity with temperature above 100 K in all samples; (e) the existence of a critical temperature (100 K) at which the coercivity is minimum; (f) the surprising suppression of coercivity upon field-cooling; and (g) the observation of coercivity at all temperatures, even at 300 K. The results are discussed and attributed to the existence of spin glass clusters at the core–shell interface.

## 1. Introduction

Magnetic nanoparticles (MNPs) are of great interest due to their energy and bio-medical applications [1,2,3,4,5,6,7,8,9], e.g., data storage systems. Coercivity and exchange bias are among the most important magnetic parameters that could influence the magnetization of MNPs. Core–shell ferromagnetic (FM)–antiferromagnetic (AFM) and ferrimagnetic (FIM)–antiferromagnetic (AFM) MNPs have drawn large attention due to their applications in permanent magnets and high-density recording media [10,11]. Shifting of the magnetization versus applied magnetic field (*M-H*) hysteresis loops along the magnetic field axis (horizontal direction) was observed in such core–shell MNPs. This shifting of the magnetization hysteresis loop is called the exchange bias effect and was attributed to exchange coupling at the core–shell interfaces [1,12,13]. Exchange bias can provide an extra source of anisotropy, leading to stable magnetization [14]. However, the origin of the exchange bias is yet to be fully understood. According to earlier reports, the exchange coupling between the FIM–AFM and FM–AFM interfaces is the source of the exchange bias [15]. A large number of studies reported and discussed the exchange bias in bilayer and multilayer thin films [16,17], nanoparticles with core–shell structures [18,19,20], and particles dispersed in matrix [21]. It was also suggested that surface effects in magnetic nanoparticles induce exchange bias [22]. Due to their small size, a large portion of atoms in nanoparticles are surface atoms [23]. The defects on the surface such as atomic vacancies, changes in the atomic coordination, dangling bonds, and lattice disorder lead to uncompensated spins contributing surface magnetization. The surface magnetization depends on the size of the nanoparticle and on the degree of surface disorder [24,25]. Usually, a magnetic nanoparticle is assumed to be made up of a single domain particle with uniaxial anisotropy. In the case of core–shell nanoparticles, the interface atoms have a different environment than those in the core of the particle. The exchange bias is suggested to be influenced by several effects, such as the area of the core–shell interface [26], the roughness of the core–shell interface [27], the thickness of the shell [28], and the competition between the magnetostatic anisotropy and the exchange coupling [27]. The magnetic properties of Fe_3_O_4_–γ-Fe_2_O_3_ core–shell nanoparticles are not well-investigated. Magnetite and maghemite are ferrimagnetic. Pure stoichiometric bulk magnetite has a saturation magnetization (MS) of 92–100 Am^2^/kg (emu/g), exhibits a Verwey transition (*T*_V_) around 120 K, and has a Curie temperature (*T*_C_) of 850 K, above which it becomes paramagnetic [29,30]. Magnetite (Fe_3_O_4_) has an inverse cubic spinel structure with an Fd3m space group and lattice constant of a = 8.396 Å. On the other hand, γ-Fe_2_O_3_ has a cubic or tetrahedral defect spinel structure with a P4_3_32 (cubic) and P4_1_2_1_2 (tetragonal) space group. It has lattice constants of *a* = 8.3474 Å for cubic, and *a* = 8.347 Å, *c* = 25.01 Å for tetragonal structure, respectively. Bulk maghemite has a saturation magnetization of 60–80 Am^2^/kg (emu/g). The determination of its Curie temperature is difficult because it is metastable at high temperature, where it transforms to hematite. However, its Curie temperature is estimated to be about 820–986 K [29,30]. There are many studies on layered and core–shell magnetic structures [1,2,31]. Recently, many studies also reported the exchange bias effect in core–shell FM–AFM nanoparticles. Most of these studies investigated the conventional exchange bias which occurs when *T*_C_ of the FM core is larger than Néel temperature (*T*_N_) of the AFM shell. Unconventional exchange bias is said to occur when *T*_C_ of the FM core is smaller than *T*_N_ of the AFM shell, or when the FM and AFM order is inverted, such that the core is AFM and the shell is FM. Some studies also reported exchange bias in FM–ferrimagnetic (FIM) and FIM–FM core–shell nanoparticles [1]. However, very few studies were conducted on FIM–FIM core–shell nanoparticles [32,33].

In Fe_3_O_4_–γ-Fe_2_O_3_ nanoparticles, the role of shell dimensions and temperature on the behavior of the coercivity and exchange bias is not well-understood. Theoretical studies on the shell-dependent exchange bias for both doubly inverted structure and normal FM–FIM core–shell structure were conducted [34,35]. However, if the *T*_N_ of the AFM is larger than *T*_C_ of the FM, the systems are usually called ‘‘doubly inverted’’, e.g., MnO/Mn_3_O_4_. For the doubly inverted system, the exchange bias was predicted to be enhanced for thicker shell when the core dimensions (~6 nm) were sufficiently small [34]. In case of FM–FIM core–shell nanoparticles, exchange bias was observed to increase monotonically and to saturate after certain shell thickness (~10 nm). When the shell thickness is reduced below 10 nm, the exchange bias was found to decrease rapidly [35]. Hence, the role of the shell thickness in the FM–FIM core–shell nanoparticle system deserves more investigations. In this study, we have investigated the role of temperature and shell thickness on the coercivity and exchange bias of Fe_3_O_4_–γ-Fe_2_O_3_ nanoparticles with nearly the same core size but with three different shell thicknesses.

## 2. Results

### 2.1. Structural and Phase Analysis

**Figure 1:**
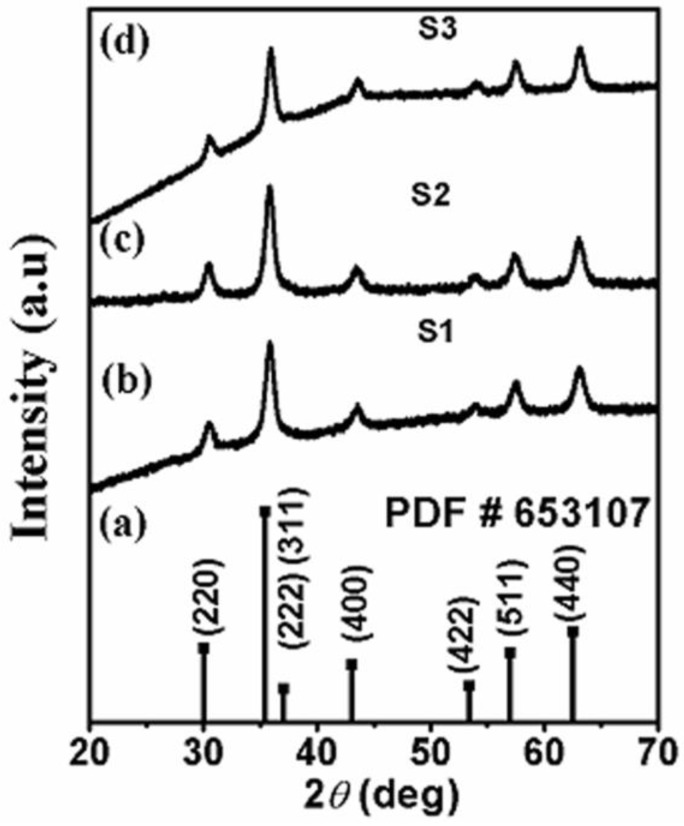
(**a**) The JCPDS data and the X-ray diffraction (XRD) patterns of Fe_3_O_4_–γ-Fe_2_O_3_ core–shell nanoparticles for samples (**b**) S1; (**c**) S2; and (**d**) S3.

The Fe_3_O_4_ nanomaterials with different particle sizes are prepared by a co-precipitation method. The phase purity of the samples was confirmed by X-ray diffraction analysis (XRD) and the pattern is shown in Figure 1 for 2*θ* ranging from 20° to 70°. All the peaks were indexed to Fe_3_O_4_ and γ-Fe_2_O_3_, and we did not see any signature of secondary phases confirming that the phase is pure. Note that the XRD peaks of Fe_3_O_4_ and γ-Fe_2_O_3_ phases were found to be in same position including high intensity peak due to (311) plane for both Fe_3_O_4_ and γ-Fe_2_O_3_. Also, extra peak intensities due to (321) and (221) lattice planes corresponding to the γ-Fe_2_O_3_ compound were not observed clearly from the XRD. The XRD peaks due to (511), (422), (311), (220), and (440) lattice planes corresponding to Fe_3_O_4_ phase were clearly visible.

The sizes of the nanoparticles were inferred from the XRD data by using Scherrer’s formula:

*D*_P_ = 0.94λ/βcos *θ*, (1)

where *D*_P_ is the average crystallite size, λ is the X-ray wavelength, β is the full width of half maximum (FWHM) of the XRD line, and *θ* is the Bragg’s angle. The average sizes calculated from Scherrer’s formula were 9, 11, and 13 nm for samples S1, S2, and S3, respectively.

### 2.2. Morphological Analysis

**Figure 2:**
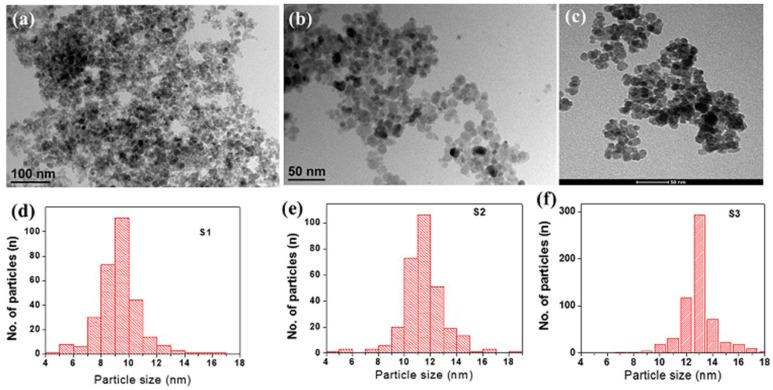
The transmission electron microscopy (TEM) images of samples (**a**) S1; (**b**) S2; and (**c**) S3. The particle size histograms of the samples (**d**) S1; (**e**) S2; and (**f**) S3.

**Figure 3:**
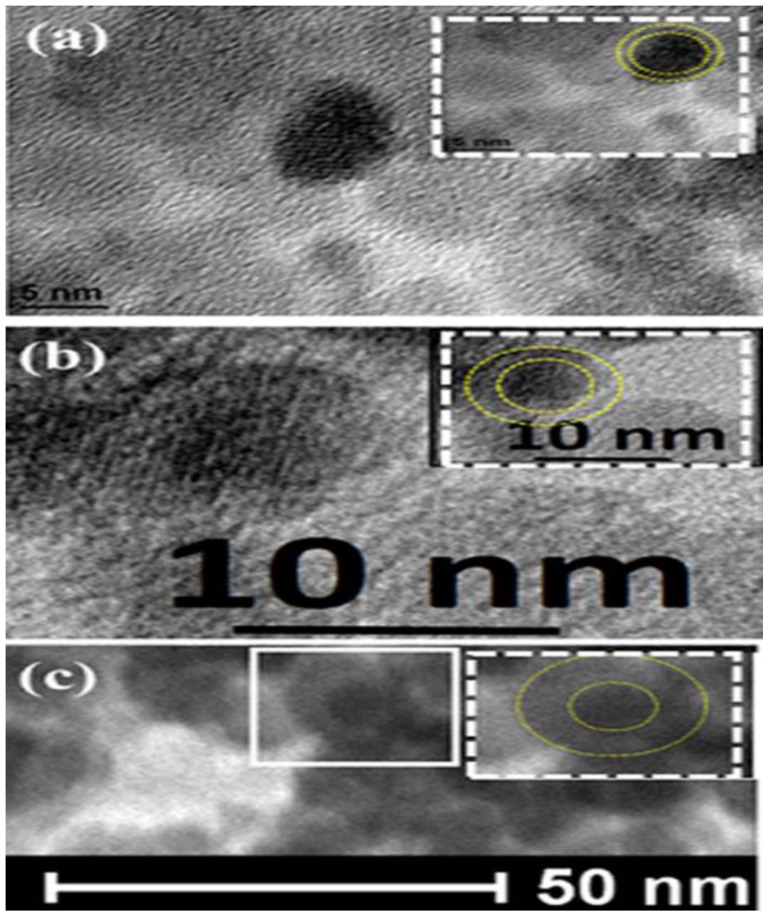
The high-resolution TEM (HRTEM) of samples (**a**) S1; (**b**) S2; and (**c**) S3. The dotted lines in the insets are guides for the eye and show the boundaries of the cores and shells.

The morphology of the phase pure samples was studied using transmission electron microscopy (TEM) and high-resolution TEM (HRTEM) techniques. Figure 2 displays the TEM images and the particle size histograms of the three samples. The TEM images show that the particles in all samples are almost spherical in shape. The particle size histograms, which were obtained from TEM measurements on many particles, show narrow size distributions with average sizes of 9, 11, and 13 nm, for samples S1, S2, and S3, respectively. Some aggregations can be observed in the TEM images. Figure 3 displays the HRTEM of all samples.

The average core and shell dimensions were obtained from several HRTEM images. The core–shell structure is well displayed where the core appears darker than the shell. The average dimensions of the cores and shells were calculated form several HRTEM images. All the samples were found to have the same average core diameter of approximately 8 nm. The average shell thicknesses for samples S1, S2, and S3 were found to be 1, 3, and 5 nm, respectively. In the insets of Figure 3, the dotted lines were drawn at the core–shell interface as well as the surface of the nanoparticle as guides for the eye.

**Figure 4:**
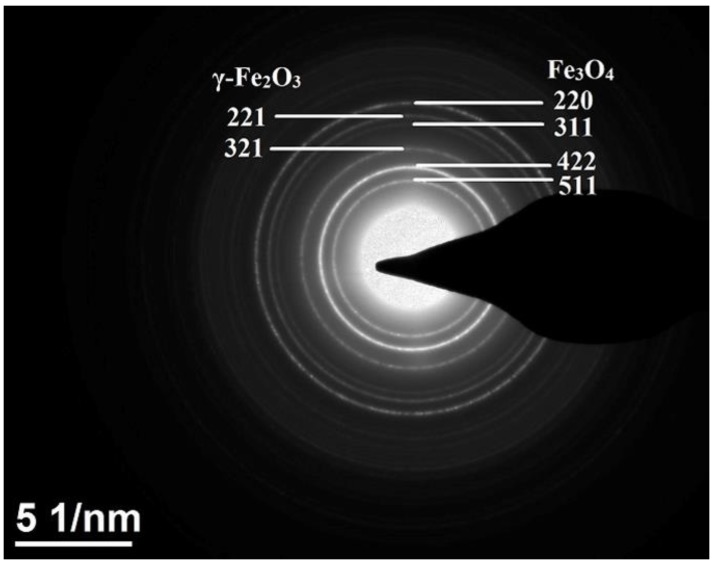
The selected area electron diffraction (SAED) pattern for sample S2.

Selected area electron diffraction (SAED) patterns as shown in Figure 4 confirmed the Fe_3_O_4_–γ-Fe_2_O_3_ core–shell structure of the nanoparticles. Figure 4 shows the SAED pattern for sample S2, which is a representative of the SAED patterns of all samples. The ring-type pattern confirms the polycrystalline nature of the sample. The figure also clearly confirms the presence of Fe_3_O_4_ and γ-Fe_2_O_3_ crystal planes forming the core–shell structure. The highest intensity peaks (311) for both Fe_3_O_4_ and γ-Fe_2_O_3_ phases were for *d* = 0.253 and 0.252 nm, respectively. Hence, the (311) plane shown in Figure 3b corresponds to the superimposed planes of both the phases. The existence of (511), (422), (311), and (220) planes of Fe_3_O_4_ corresponding to *d* = 0.161 nm, 0.171 nm, 0.253 nm, and 0.296 nm, respectively, are confirmed. The presence of the (321) and (221) planes of γ-Fe_2_O_3_ corresponding to the *d* = 0.253 and 0.278 nm, respectively, are verified as pointed out in the figure. This clearly confirmed that the core–shell nanoparticles are Fe_3_O_4_/γ-Fe_2_O_3_ nanoparticles.

### 2.3. Mössbauer Analysis

**Figure 5:**
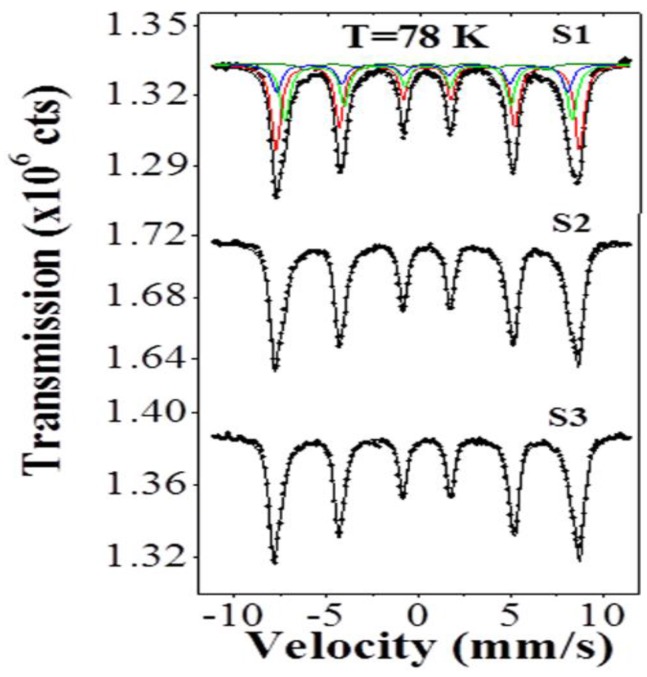
Mössbauer spectra, at 78 K, of the three different nanoparticle samples (the solid curves represent the fitting). The subspectra in the top of the sample labeled S1 represent the detected components. The other samples have similar subspectra.

Figure 5 shows the Mössbauer spectra (dots) at 78 K for the different samples, and the fitting is represented by the solid curves. It is clear from this figure that the spectra of all the samples show magnetically split components. The spectra are fitted with four six-line patterns: one component for the γ-Fe_2_O_3_ (ferromagnetic), two components (with ratios of 2:1 for the Octahedral and the Tetrahedral sites) for the Fe_3_O_4_ (Ferrimagnetic), and the fourth one with very small percentage, which might be due to the interface. Table 1 presents the Mössbauer parameters for the different components without the interface, which has the values of *δ* = 0.37 mm/s, *B*_hf_ = 43 T, *w* = 0.50–0.54 mm/s, and very small relative intensity of *A*= 2.3–3.3%. One aim of the Mössbauer study is to determine the percentages of γ-Fe_2_O_3_ and Fe_3_O_4_, which can be done using the percentage area (relative intensity) of each subspectra. The isomer shift (δ) and the hyperfine magnetic field (*B*_hf_) are the main parameters which are used to distinguish between the γ-Fe_2_O_3_ and Fe_3_O_4_ in the Mössbauer spectra. Our values for δ and B_hf_ are in good agreement with the previously published values of *δ* = 0.42–0.44 mm/s and *B*_hf_ = 50–51 T for γ-Fe_2_O_3_, and for Fe_3_O_4_ *δ* = 0.46–0.50 mm/s and *B*_hf_ = 47.0–48.5 T for the Octahedral site and *δ* = 0.23–0.25 mm/s and *B*_hf_ = 49–50 T for the Tetrahedral site [36,37,38,39,40,41,42].

From the density of 5.24 g/cm^3^ and the molar mass of 159.688 g/mol for γ-Fe_2_O_3_, and from the density of 5.17 g/cm^3^ and the molar mass of 231.533 g/mol for Fe_3_O_4_, we can estimate the percentage of iron (or the number of Fe-atoms) in equal volumes of γ-Fe_2_O_3_ and Fe_3_O_4_. By dividing the molar mass over the density for γ-Fe_2_O_3_, we get 32.81 × 10^−3^ mol/cm^3^, and for Fe_3_O_4_ we get 22.32 × 10^−3^ mol/cm^3^, since the mol of the material contains Avogadro’s number (N_A_) of the molecules; then for γ-Fe_2_O_3_ we get 2 × 32.81 × 10^−3^ = 65.63 × 10^−3^ of N_A_ Fe-atoms/cm^3^, and for Fe_3_O_4_ we get 3 × 22.32 × 10^−3^ = 66.9 × 10^−3^ of N_A_ Fe-atoms/cm^3^. These values show that the number of Fe-atoms in the same volume of γ-Fe_2_O_3_ and Fe_3_O_4_ is about the same (i.e., if we divide 65.63 by 66.9 we get the ratio of 0.981). Accordingly, from the percentage area (relative intensity) of each subspectra of the Mössbauer spectra, we can find the ratio of the γ-Fe_2_O_3_ to that of Fe_3_O_4_ in each sample. Table 2 shows these ratios and the calculated ratio for the volumes occupied by Fe_3_O_4_ in the core and for γ-Fe_2_O_3_ in the shell. It can be seen from the last two columns of Table 2 that these ratios are almost the same, which confirms the occupancy of Fe_3_O_4_ in the core and γ-Fe_2_O_3_ in the shell.

From this Mossbauer data we confirm that the ratio of the γ-Fe_2_O_3_ phase is equal to the shell-to-core volumes ratio regardless of the shell thickness. Thus, the core and shell phases do not change with the change of shell thickness.

### 2.4. Magnetic Properties

Magnetization versus applied magnetic field hysteresis loops (*M*-*H*) were measured at several temperature values, 2, 50, 100, 200, and 300 K. In each loop cycle, the magnetization was recorded while the applied field was changing between the maximum values of −3 T and 3 T. In the field-cooled (FC) protocol, for each loop measurement, the temperature of the sample was brought to room temperature followed by the application of a magnetic field of a specific value (the FC value). The sample was then cooled down from room temperature to one of the above mentioned temperatures while the field remains applied. At each temperature, the *M*-*H* was measured at the FC values *H*_FC_ of 0.5, 1, 2, and 3 T, respectively.

**Figure 6:**
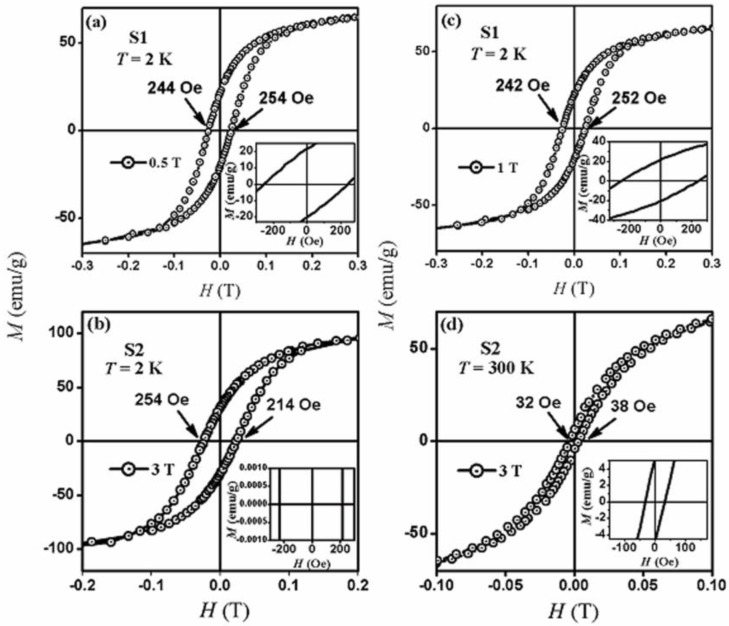
Magnetization versus applied magnetic field (*M*-*H*) hysteresis loops for (**a**) sample S1 at 2 K and at *H*_FC_ = 0.5 T; (**b**) sample S2 at 2 K and at *H*_FC_ = 3 T; (**c**) sample S1 at 2 K and at *H*_FC_ = 1 T; and (**d**) sample S2 at 300 K and at *H*_FC_ = 3 T. The insets are enlargements of the region around the origin.

Selected *M*-*H* hysteresis loops for samples S1 and S2 are shown in Figure 6. For sample S1, the *M*-*H* hysteresis loops are shown at 2 K at field-cooling values of 0.5 and 1 T. For sample S2, the *M*-*H* hysteresis loops are shown at field cooling value of 3 T at 2 and 300 K. Typical hysteresis loops were obtained at other temperatures and *H*_FC_ values for all samples.

From the insets of Figure 6, it was observed that both samples display horizontal loop shifting. The horizontal shift of the loops was defined as the exchange bias field, *H*_EB_. From these magnetization hysteresis loops, the exchange bias field, *H*_EB_, and the coercivity, *H*_C_, were calculated for all samples at several conditions. *H*_C_ and *H*_EB_ were calculated using the formulas:

*H*_EB_ = (*H*_C1_ + *H*_C2_)/2 (2)

**Figure 7:**
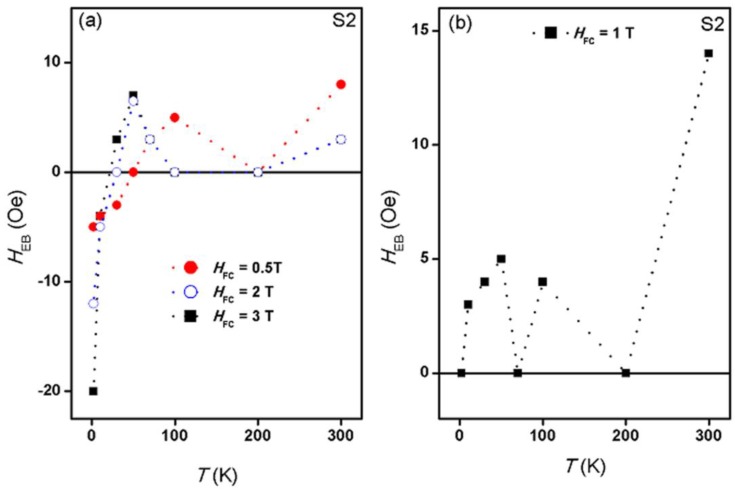
The temperature dependence of *H*_EB_ in the temperature range of 2–300 K for sample S2 under (**a**) *H*_FC_ = 0.5, 2, 3 T; and (**b**) *H*_FC_ = 1 T.

**Figure 8:**
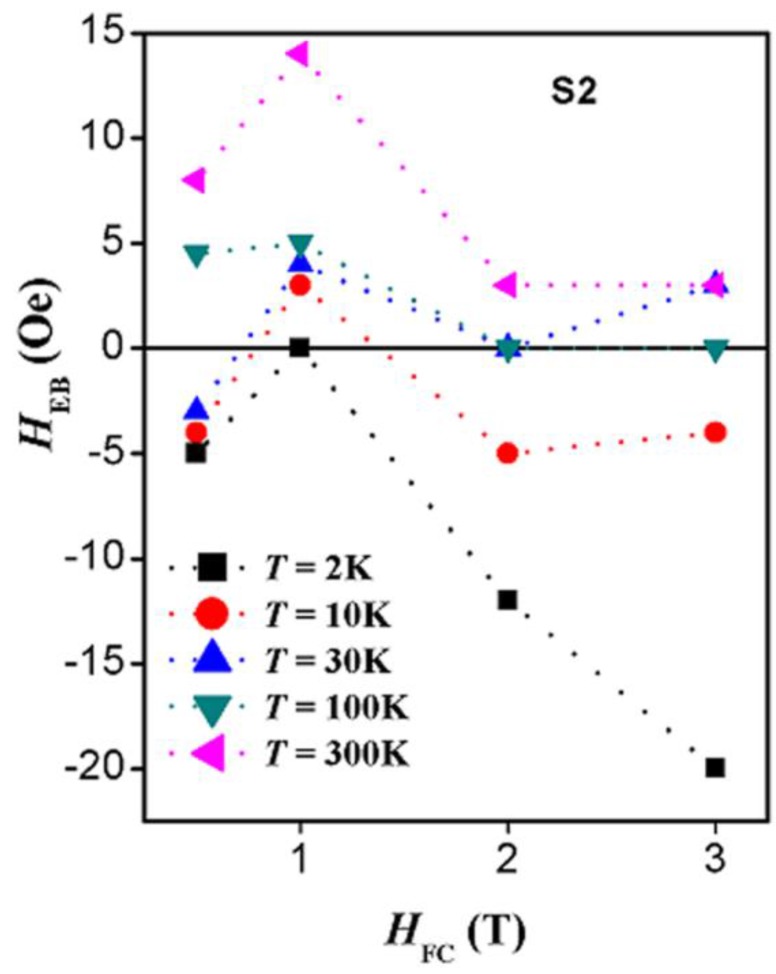
The field-cooling dependence of *H*_EB_ in sample S2 at several temperatures.

*H*_C_ = (*H*_C2_ − *H*_C1_)/2 (3)

where *H*_C1_ and *H*_C2_ are the coercive fields (including their signs) at the descending and the ascending branches of the *M-H* hysteresis loop, respectively. Figure 7 shows the exchange bias field versus temperature in sample S2 at several field-cooling values. It can be seen in this figure that at very low temperatures (below 50 K), *H*_EB_ is negative with an absolute value at 2 K that increases with *H*_FC_. This absolute value decreases with temperature up to a temperature *T*_0_, at which it becomes zero. Above *T*_0_, *H*_EB_ becomes positive and increases until it reaches a maximum at a temperature *T*_M_. Above *T*_M_, *H*_EB_ decreases until it becomes zero again at the blocking temperature, *T*_B_ (which is 100 or 200 K, depending on the *H*_FC_). Above *T*_B_, *H*_EB_ becomes positive and increases up to 300 K. A similar trend of the temperature dependence of *H*_EB_ up to 200 K in our samples was reported in spin-glass-ferromagnet bilayers [43] under several *H*_FC_. This trend was also reported in polycrystalline Fe–Cr bilayers [44] under only one field-cooling value of *H*_FC_ = 1 kOe. The temperature-driven sign reversal of *H*_EB_ was attributed to Fe–Cr spin glass. In [43], *T*_0_ and *T*_M_ were found to decrease with increasing *H*_FC_, whereas *H*_EB_ at *T*_M_ was found to increase. These observations are almost similar to what we have observed in our core–shell nanoparticle system. However, there are some discrepancies, where in their spin-glass-ferromagnet bilayers system, *H*_EB_ at 2 K decreased with increasing *H*_FC_ and *T*_M_ increased with increasing *H*_FC_. These differences could be due to the different systems studied. The sign change of *H*_EB_ was also observed in a ferromagnet–spin-glass Co–CuMn bilayer system [45]. Up to our knowledge, such sign-change in *H*_EB_ has not been reported in core–shell nanoparticles. The similarity of the temperature dependence of *H*_EB_ in our sample S2 with those reported in layered structures which include spin-glass (SG) hints for the SG phase in our samples. At *H*_FC_ = 1 T, *H*_EB_ does not change its positive sign but oscillates with temperature. In our core–shell nanoparticles, the temperature dependence of *H*_EB_ strongly depends on field-cooling. This point is clearly seen in Figure 8, which displays the field-cooling dependence of *H*_EB_ at several temperatures. It is worth mentioning that *H*_EB_ has a peak value at 1 T at almost all of the temperatures.

At zero-field-cooling, no exchange bias was observed in all samples at all temperatures. After field-cooling, the main results (as displayed in Figure 7 and Figure 8) are as follows. (a) The complete disappearance of the exchange bias effect in the sample S3 at all field-cooling values and at all temperatures. (b) The occurrence of unconventional exchange bias in samples S1 (with a maximum value of 5 Oe) only at 2 K and at 10 K at particular field-cooling values. At 2 K, the exchange bias was observed only at the smallest field-cooled values of 0.5 T, and 1 T whereas at 10 K, it was observed only at 0.5 T. (c) The occurrence of unconventional exchange bias in samples S2 (with a maximum value of 20 Oe) at all temperatures and all field-cooling values. The exchange bias effect appears after field-cooling from room temperature (300 K) which is much lower than both the *T*c of the shell (which is above 820 K) and the *T*_C_ of the core (which is around 850 K) [46,47]. (d) The exchange bias in sample S2 has a nonmonotonic behavior with temperature as well as with field-cooling. This is opposite to the familiar exchange bias behavior in other systems where exchange bias decreases with temperature and increases with the increase of the field-cooling value. (e) The exchange bias in sample S2 did not vanish at 300 K at all field-cooled values. (f) Negative as well as positive exchange bias were observed in sample S2. The switch from negative to positive exchange bias in this sample occurred at temperature of 50, 30, and 21 K, for field-cooling values of 0.5, 2, and 3 T, respectively. Hence, we can state that in this FIM–FIM core–shell structure, a shell thickness of 3 nm represents a critical thickness at which the exchange bias occurs nearly at all temperatures and at all field-cooling values. In addition, 1 T is a critical field-cooling value in sample S2, where only positive exchange bias occurs and the exchange bias displays a peak or a dip at nearly all temperatures.

**Figure 9:**
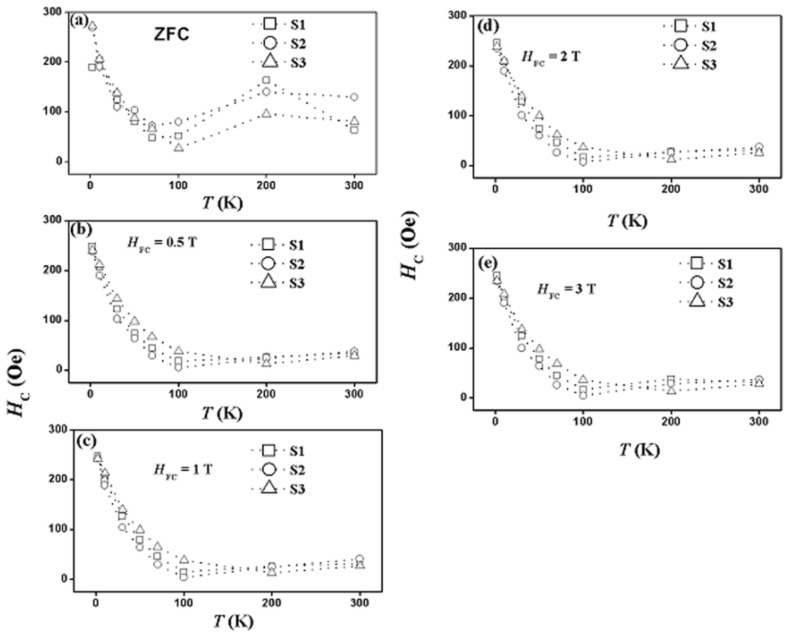
The coercivity as function of temperature for all samples in the zero-field-cooling (ZFC) state and in the field-cooled (FC) state at several field-cooling values.

**Figure 10:**
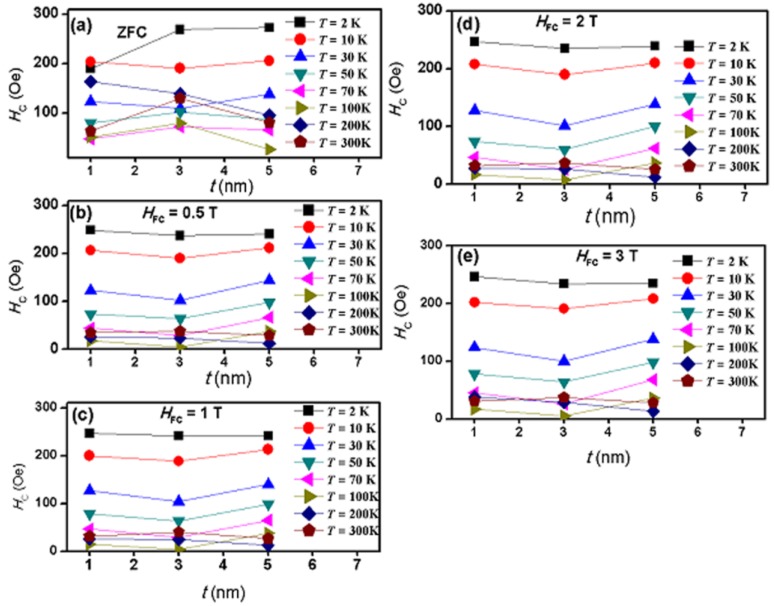
The coercivity as function of shell thickness at several temperatures in the ZFC state and in the FC state at several field-cooling values.

Figure 9 shows the coercivity as a function of temperature for all samples in the zero-field-cooling (ZFC) state and in the FC state at several field-cooling values. Figure 10 shows the coercivity as a function of shell thickness at several temperatures in the ZFC state and in the FC state at several field-cooling values.

The key results displayed by these two figures are as follows. (a) Coercivity, with appreciable magnitudes, has been detected in all samples at all temperatures at zero-field-cooling as well as at all field-cooling values. (b) The coercivity in all samples behaves in a systematic manner with temperature at zero-field-cooling as well as at all field-cooling values. (c) As function of temperature, coercivity in all samples (at zero-field-cooling and at all field-cooling values) was observed to initially decrease monotonically and sharply with increasing temperature, reaching a non-zero minimum value at 100 K for samples S1 and S2 and at 200 K for sample S3. (d) As the temperature increases above 100 K (for samples S1 and S2) and above 200 K (for sample S3), a surprising and interesting slow (and almost linear) increase of coercivity with temperature takes place and continues up to 300 K. (e) After field cooling, coercivity displayed the same behavior but with different magnitudes. The increase of coercivity with temperature after reaching a minimum value and the non-vanishing of coercivity at room temperature in all samples (at zero-field-cooling and at all field-cooled values) are novel and very important observations in this FIM–FIM core–shell structure. This behavior of coercivity is opposite to that in other systems where coercivity keeps decreasing with temperature. (f) In the zero-field-cooled state (at a particular temperature), the shell thickness has a noticeable role on coercivity, where it increased at some temperatures and decreased at others. (g) In the field-cooled state (at particular field-cooling value and temperature), shell thickness does not have any noticeable effect on coercivity. (h) The largest coercivity measured in our study occurred in sample S2 at 2 K in the zero-field-cooling state. (i) At zero-field-cooling, the magnitude of coercivity in each sample is the largest at 2 K. (j) After field-cooling at 0.5 T, the magnitudes of coercivity decrease in all samples at all temperatures (except at 2 K in sample S1). No significant additional changes in the magnitudes of coercivity occurred at higher field-cooling values in all the samples. However, after field-cooling, the magnitude of coercivity in each sample remains the largest at 2 K. Hence, field-cooling has a negative effect on coercivity in all samples and at all temperatures. This behavior of coercivity is opposite to that in other systems, where coercivity increases with the increase of the field-cooling values. (k) In sample S1, the field-cooling value of 0.5 T enhances coercivity at 2 K. Larger field-cooled values showed no significant additional enhancement. (l) At temperatures below 100 K (at zero-field-cooling as well as after field-cooling), the magnitudes of coercivity are different in different samples. (m) At temperatures above 100 K, the magnitudes of coercivity are nearly the same in all samples in the field-cooled state, whereas they are different in different samples in the zero-field-cooled state. (n) At zero-field-cooling, the rate of decrease of coercivity with temperature (below 100 K) has different values in different samples, where it is the slowest in sample S1 and the fastest in sample S3. (o) After field-cooling at 0.5 T, the rate of decrease of coercivity with temperature (below 100 K) becomes nearly the same in all samples. No observable additional changes of this behavior occurred at higher field-cooling values. (p) At zero-field-cooling, the rate of increase of coercivity with temperature (above 100 K) has different values in different samples. (q) After field-cooling at 0.5 T, the rate of increase of coercivity with temperature (above 100 K) becomes nearly the same in all samples. No significant additional changes in the behavior of coercivity occurred at larger field-cooling values. (r) Although in the field-cooled state, the rate of increase of coercivity with temperature (above 100 K) is nearly the same in all samples, it is slower than that in the zero-field-cooled case. Hence, we can consider 0.5 T as a critical field-cooling value at which coercivity starts decreasing in all samples at all temperatures.

## 3. Discussion

The existence of the exchange bias effect in some samples at some conditions after field-cooling from 300 K, which is much lower than the *T*_C_ of the shell (which is above 820 K) and the *T*_C_ of the core (which is around 850 K) [1,17], is unconventional. In systems where the shell material was AFM and field-cooling occurred from temperatures below the *T*_N_ of the shell material, this behavior was attributed to the spontaneous exchange bias mechanism [18,19].

We believe that the Fe_3_O_4_ core and γ-Fe_2_O_3_ shell materials are not in good contact due to large interface defect concentration. This can be seen in HRTEM images where the interface is not well-ordered. The interface defects could result in large interface strain, which lowers the interfacial exchange coupling and thus lowers the exchange bias field. The interface defects also could lead to a magnetic disorder at the interface, which results in the existence of interfacial uncompensated spins. These interfacial spins could freeze at low temperatures causing spin-glass clusters (SGCs) with random spin orientations. In addition, the spin-glass phase could exist at the core–shell interface due to interfacial canted spins. It is well-established that the spin–orbit coupling is responsible for the magnetocrystalline anisotropy. Lattice parameters and distortion (which decrease symmetry) have significant influence on the magnetocrystalline anisotropy. Broken exchange bonds at the surface result in the reduced coordination of surface cations, which leads to changes in their spin–orbit energy, resulting in changes in the local magnetocrystalline anisotropy [20,21]. In addition, low symmetry near the surface produces large contribution to the local magnetic anisotropy, resulting in spin canting [22]. This is the expected case also at the core–shell interface where these interfacial canted spins can freeze into SGCs. Randomly spread spin-glass-like phases at the layered FM–AFM and core–shell nanoparticle interfaces were proposed to significantly influence the exchange bias properties [1,23,24]. The interfacial strain also could lead to a spin-glass phase. Because of the small size of nanoparticles, the radius of curvature is very small. For core–shell nanoparticles, lattice mismatching between the shell and core materials is expected. Hence, a large degree of interfacial structural disorder is expected to occur in such nanoparticle systems. The smaller the core diameter, the larger is the interfacial structural disorder [7]. Although the core was fixed at 8 nm in our core–shell nanoparticles, the shell thickness was varied, which results in varied stress values on core leading to different interfacial defects and, hence, different quantities of spin-glass clusters. In addition, it was reported that surface spins in γ-Fe_2_O_3_ nanoparticles are more magnetically disordered than in Fe_3_O_4_ nanoparticles of the same size [25,26,27], and thus we expect larger amount of interfacial spin-glass as the shell thickness increases.

The interfacial SGCs prevent the direct exchange coupling of the core and shell spins. Each of these SGCs has a fixed direction, which is the direction of the net spins composing the cluster. The directions of net spins of all clusters are randomly oriented. Their temperature behavior is not well-defined because of the unknown pining strengths and orientations of these spins.

We suggest that the interfacial spin-glass phase is composed of several SGCs, where each SGC has a net spin (magnetic moment) and the spins in the SGCs are randomly oriented. These SGCs are coupled to each other and to the core and shell surfaces. The pinning strength of the spin of each SGC is determined by its exchange interactions with nearby spins (of other SGCs), the core spins, and shell spins near the interface. At high temperatures, these SGC spins are weakly coupled and can be considered as free. At low temperatures, they freeze in random directions with strong couplings.

Several signatures of the spin-glass behavior in these core–shell nanoparticles were obtained (and will be reported in a separate paper). Hence, this model of interpretation could be reasonable for our samples and other core–shell nanoparticles with similar core–shell materials and similar interfacial structure. For core–shell nanoparticles with good core–shell contact, less (or no) concentrations of interfacial defects might occur with the absence of interfacial SGCs, leading to a different behavior of exchange bias field and coercivity with temperature and field cooling. It is worth mentioning that the amount of the interfacial SGCs can be experimentally determined using X-ray magnetic circular dichroism (XMCD) [28,46,47]. Another method that was applied to layered FM–AFM structures uses the so-called blocking temperature distribution from magnetic hysteresis loops at several conditions [34,35]. We have not conducted any study on quantifying the SG phase in our core–shell nanoparticles.

In FM–AFM core–shell nanoparticles, *H*_EB_ was shown to increase with increasing the field-cooling value and then saturates for field-cooling values above 2 T [48]. This behavior was attributed to the polarization of the uncompensated spins as the cooling field is increased. In [49], exchange bias with small values was observed in very small FeO nanoparticles of average diameters of 3.3 nm. The occurrence of exchange bias in these nanoparticles was attributed to the limited existence of antiferromagnetically compensated spins with in each particle. In FM–AFM systems involving spin-glass-like behavior or spin disorder, with increasing the field-cooling value, *H*_EB_ decreased after reaching a maximum without saturation [15,35,50]. Our exchange bias results for sample S2 do not contain any of these behaviors. The common observation in our data is that *H*_EB_ increases with increasing the field-cooling value reaching a maximum and then decreases. With further increase of field-cooling value, *H*_EB_ keeps decreasing, reaches saturation, or increases slightly.

The nearly similar magnitudes of coercivity along with its systematic behavior with temperature in all samples at all field-cooled values are clear indications of the insignificant role of shell thickness on coercivity. Hence, it might be concluded that the coercivity in our core–shell nanoparticles is mainly determined by core effects and the exchange coupling between the core spins near the interface and the interfacial SGCs. The different behavior of coercivity with temperature in these samples was displayed only in the zero-field-cooled case, where slightly different magnitudes and different rates of decrease (below 100 K) or increase (above 100 K) are observed in different samples. At temperatures below 100 K, the fast and monotonic decrease of coercivity with temperature in all samples is attributed to the thermal activation effects of the blocked core moments (spins) over the anisotropy barriers. However, the different rates of decrease of coercivity with temperature (below 100 K) is not a core effect. Because all these particles have nearly the same core size, we believe that this different behavior in coercivity is due to the different nature of the interfacial SGCs. A field-cooled value of 0.5 T is large enough to align the spins of each cluster and thus eliminate the different identities of each SGC in each sample. In the field-cooling process, the spins within each SGC will be aligned in the same direction and thus behave similarly. The temperature provides the interfacial SGCs with the energy needed to unpin the spins within each cluster and hence decrease the exchange coupling with the nearby core spins, regardless of the initial field-cooling. The small and linear increase of coercivity above the critical temperature could be attributed to an enhanced interaction between the core spins near the interface and the interfacial spin-glass clusters. As the temperature is raised above 100 K, the SGC will start to vanish and the initially pinned spins within each cluster will start to become unpinned and rotate in the direction of the applied magnetic field. Hence, an enhanced exchange coupling starts to occur between these well-directed spins and the nearby core spins, leading to the small increase in coercivity with temperature.

The different behaviors of coercivity and exchange bias with temperatures at all field-cooled values hint for different mechanisms of interaction of these effects in such FIM–FIM core–shell structure. The variations of exchange bias with shell thickness is a clear indication on the role of the whole shell material on exchange bias. The nonmonotonic behavior of exchange bias with temperature in sample S2 (especially the enhanced exchange bias at 300 K) is an indication of the role of the interfacial SGCs. Hence, we propose that the exchange bias effect in our core–shell nanoparticles is mainly due to the indirect exchange coupling between the core and shell spins. The layer of interfacial SGCs mediates the exchange coupling between the core and shell spins. The exchange bias vanishes in the sample S3 (with the thickest shell) because the shell spins are under-coupled, and the samples behave as if they do not possess shells. The vanishing of exchange bias (in most conditions) in sample S1 (with the thinnest shell) occurs because the core and shell spins are over-coupled, causing the shell spins to be completely locked to the core spins and rotate with them, and hence giving no exchange bias. The core and shell spins in sample S2 are coupled enough such that the rotation of the core spins will be opposed by the shell spins, producing exchange bias effect. Temperature and an applied magnetic field might influence the spin-glass interfacial clusters in a particular region more than in others, depending on the strength of spin coupling (pinning) within these SGCs and their coupling with the core and shell spins. The different and unpredicted responses of the spin-glass interfacial clusters to the temperature and applied field lead to unpredicted temperature and field dependence of the indirect exchange coupling between the core and shell spins, and hence of the exchange bias as observed in sample S2. The small exchange bias magnitudes in all samples could be attributed to the weak exchange coupling between the core and shell spins that is mediated by the interfacial SGCs. The nonmonotonic behavior attributed to the unpredicted behavior of these spin-glass clusters with temperature and field-cooling. The enhanced exchange bias at 300 K could be attributed to the enhanced indirect core–shell coupling due to the directed spins in these clusters. The positive exchange bias could be due to an antiferromagnetic nature of the exchange interaction at the interface, whereas the negative exchange bias could be due to a ferromagnetic nature of the exchange interaction at the interface.

## 4. Materials and Methods

The key and novel result here is the successful control of the core and shell dimensions, where the core diameter was fixed at nearly 8 nm while the shell thickness was varied from 1 to 5 nm. We have synthesized iron oxide (Fe_3_O_4_–γ-Fe_2_O_3_) nanomaterials with different particle sizes by the usual co-precipitation method. The FeCl_3_·6H_2_O (0.1 M) and FeCl_2_·4H_2_O (0.05 M) were used as precursor materials. Both the precursor materials were dissolved in deionized water separately and mixed at room temperature. Ammonium hydroxide solution (25%) was added drop by drop until PH became 10. The mixed solution was kept at 80 °C for the reaction to occur. The reaction was carried out in atmospheric pressure, and the reaction times were 1, 2, and 3 h to get nanoparticles with different sizes. Finally, a black precipitation was formed, collected, and dried at 120 °C in vacuum. It was reported that a reaction mechanism involving inward anionic diffusion was responsible for the γ-Fe_2_O_3_ phase [51]. The atomic oxygen plays a major role in inducing the γ-Fe_2_O_3_ phase. This is due to the direct logarithmic kinetic diffusion with a logarithmic relation between γ-Fe_2_O_3_ thicknesses versus oxygen exposure time. Hence, instead of keeping the nanoparticles in vacuum, they were allowed to oxidize in ambient atmosphere for different time spans. The smaller particles are allowed to oxidize in oxygen atmosphere for shorter time, and the largest particles are allowed to oxidize for longer time to get thicker shells with the same core dimensions. The nanoparticle with 1 h reaction time in open air had the smallest γ-Fe_2_O_3_ shell thickness, whereas nanoparticles with 3 h reaction time in open air had the largest γ-Fe_2_O_3_ shell thickness. All the samples were found to have the same core diameter of 8 nm. The samples, which were prepared with reaction times of 1, 2, and 3 h, were found to have shell thickness of 1 nm (sample S1), 3 nm (sample S2), and 5 nm (sample S3), respectively.

In order to confirm the corresponding phase, X-ray diffraction characterization was performed using a PANalytical X’Pert Pro X-ray diffractometer (Almelo, The Netherlands). The morphology of the nanoparticles was confirmed by several microscopic tools such as high-resolution transmission electron microscopy (HRTEM) and selected area electron diffraction (SAED). The transmission electron microscopy (TEM) measurements were performed using an FEI, TECNAI G^2^ F30, S-TWIN microscope GG Eindhoven, The Netherlands) operating at 300 kV equipped with a GATAN Orius SC1000B CCD camera. Mössbauer spectra were collected using a standard constant acceleration Mössbauer spectrometer (Wissel, Stamberg, Germany) over 1024 channels. The samples for Mössbauer studies were circular disks of diameter 1.3 cm, prepared by sprinkling a thin layer of the finely powdered alloys on a piece of Scotch tape. The γ-ray source was a 50 mCi Co^57^ in a rhodium matrix. Isomer shifts were measured relative to the centroid of the α-iron spectrum at room temperature, and α-iron spectrum was also used for calibration. The magnetic measurements were conducted using a superconducting quantum interference device (SQUID) (San Diego, CA, USA) from Quantum Design. Magnetic moment versus applied field up to 3 T was measured for the three samples at several temperatures after cooling the samples in zero field and in several applied fields.

## 5. Conclusions

The Fe_3_O_4_–γ-Fe_2_O_3_ core–shell nanoparticles with fixed core diameter and three different shell thicknesses were prepared by the usual chemical precipitation method. The X-ray diffraction and transmission electron microscopy images showed that particles are nearly spherical with diameters of 9, 11, and 13 nm, respectively. The Mossbauer spectroscopy and selected area electron diffraction ascertained that the core–shell particles mainly consisted of Fe_3_O_4_ core and γ-Fe_2_O_3_ shell. Several key results on exchange bias and coercivity were obtained and attributed to the existence of spin glass clusters at the core–shell interface. These interfacial spin glass clusters were suggested to mediate the exchange interaction between the core and shell spins.

## Figures and Tables

**Table 1:** The Mössbauer parameters at 78 K for the three different nanoparticle samples, without the interface. B_hf_ = hyperfine magnetic field in (T ± 0.1), δ = isomer shift in (mm/s ± 0.01), *A* = percentage area (relative intensity) in (% ± 0.5). The quadrupole shifts (=2ε) for all the samples were very small 2ε= 0.02 ± 0.01 (mm/s). The ratio of the line widths for the first three lines relative to the line width of the inner lines (W in (mm/s ± 0.02)) of each subspectra were fixed to the values of 1.2, 1.1, 1 in all the samples.

Sample	*B*_hf_ (T) γ-Fe_2_O_3_	*δ* (mm/s) γ-Fe_2_O_3_	*W* (mm/s) γ-Fe_2_O_3_	*A* (%) γ-Fe_2_O_3_	*B*_hf_ (T) Fe_3_O_4_	*δ* (mm/s) Fe_3_O_4_	*W* (mm/s) Fe_3_O_4_	*A* (%) of the 2-Sites Fe_3_O_4_	Total *A* (%) Fe_3_O_4_
S1	51.2	0.44	0.54	48.4	48.4 49.0	0.47 0.24	0.54 0.54	32.2 16.1	48.3
S2	51.1	0.44	0.48	80.1	48.4 49.0	0.47 0.24	0.48 0.48	11.2 5.6	16.8
S3	51.0	0.44	0.48	89.0	48.4 49.0	0.47 0.24	0.48 0.48	5.7 2.8	8.5

**Table 2:** The ratios of Shell volume/Core volume for the three different nanoparticle samples, and the ratios of the Mössbauer percentage area (relative intensity) of each subspectra [*A* (%) γ-Fe_2_O_3_/*A* (%) Fe_3_O_4_] multiplied by the factor 0.981 for the ratio of the concentration of Fe-atoms per unit volume in γ-Fe_2_O_3_ and Fe_3_O_4_.

Sample	Core Diameter (nm) Fe_3_O_4_	Shell Thickness (nm) γ-Fe_2_O_3_	Core Volume (nm^3^) Fe_3_O_4_	Shell Volume (nm^3^)γ-Fe_2_O_3_	Total *A* (%) Fe_3_O_4_	*A* (%) γ-Fe_2_O_3_	Shell Volume/Core Volume	[*A* (%) γ-Fe_2_O_3_/Total *A* (%) Fe_3_O_4_] × 0.981
S1	8±0.9	1±0.06	268.1	255.5	48.3	48.4	0.953	0.983
S2	8±0.9	3±0.05	268.1	1169	16.8	80.1	4.360	4.678
S3	8±0.9	5±0.06	268.1	2786	8.5	89.0	10.392	10.272

## References

[1] Nogues J., Sort J., Langlais V., Skumryev V., Surinach S., Munoz J.S., Baro M.D. (2015). Exchange bias in nanostructures. Phys. Rep..

[2] López-Ortega A., Estrader M., Salazar-Alvarez G., Rocad A.G., Nogués J. (2015). Applications of exchange coupled bi-magnetic hard/soft and soft/hard magnetic core/shell nanoparticles. Phys. Rep..

[3] Skumryev V., Stoyanov S., Zhang Y., Hadjipanayis G., Givord D., Nogués J. (2003). Beating the superparamagnetic limit with exchange bias. Nature.

[4] Mishra D.K. (2011). FeRh-FePt Core Shell Nanostructure for Ultra-High Density Storage Media. U.S. Patent.

[5] Liu X.S., Gu B.X., Zhong W., Jiang H.Y., Du Y.W. (2003). Ferromagnetic/antiferromagnetic exchange coupling in SrFe_12_O_19_/CoO composites. Appl. Phys. A.

[6] Groza I., Morel R., Brenac A., Beigne C., Notin L. (2011). Electrical and Magnetic Properties of Co/CoO Core–Shell Clusters. IEEE Trans. Magn..

[7] Rostamnejadi A., Venkatesan M., Alaria J., Boese M., Kameli P., Salamati H., Coey J.M.D. (2011). Conventional and inverse magnetocaloric effects in La_0.45_Sr_0.55_MnO_3_ Nanoparticles. J. Appl. Phys..

[8] Barbic M., Scherer A. (2005). Magnetic nanostructures as amplifiers of transverse fields in magnetic resonance. Sol. State Nucl. Magn. Res..

[9] Jeon S.L., Chae M.K., Jang E.J., Lee C. (2013). Cleaved Iron Oxide Nanoparticles as *T*_2_ Contrast Agents for Magnetic Resonance Imaging. Chem. Eur. J..

[10] Sort J., Nogues J., Surinach S., Munoz J.S., Baro M.D., Chappel E., Dupont F., Chouteau G. (2001). Coercivity and squareness enhancement in ball-milled hard magnetic–antiferromagnetic composites. Appl. Phys. Lett..

[11] Gangopadhyay S., Hadjipanayis G.C., Sorensen C.M., Klabunde K.J. (1992). Magnetic properties of ultrafine Co particles. IEEE Trans. Magn..

[12] Meikleohn W.H., Bean C.P. (1956). New Magnetic Anisotropy. Phys. Rev..

[13] Berkowitz A.E., Takano K. (1999). Exchange anisotropy—A review. J. Magn. Magn. Mater..

[14] Liu K., Nogues J., Leighton C., Masuda H., Nishio K., Roshchin I.V., Schuller I.K. (2002). Fabrication and thermal stability of arrays of Fe nanodots. Appl. Phys. Lett..

[15] Tang Y.-K., Sun Y., Heng Z.-H. (2006). Cooling field dependence of exchange bias in phase-separated La_0.88_Sr_0.12_CoO_3_. J. Appl. Phys..

[16] Binek C., He X., Polisetty S. (2005). Temperature dependence of the training effect in a Co/CoO exchange-bias layer. Phys. Rev. B.

[17] Yi J.B., Ding J. (2006). Exchange coupling in CoO–Co bilayer. J. Magn. Magn. Mater..

[18] Salazar-Alvarez G., Sort J., Surinach S., Baro M.D., Nogues J. (2007). Synthesis and Size-Dependent Exchange Bias in Inverted Core−Shell MnO|Mn_3_O_4_ Nanoparticles. J. Am. Chem. Soc..

[19] Kavich D.W., Dickerson J.H., Mahajan S.V., Hasan S.A., Park J.H. (2008). Exchange bias of singly inverted FeO/Fe_3_O_4_ core-shell nanocrystals. Phys. Rev. B.

[20] Hajra P., Basu S., Dutta S., Brahma P., Chakravorty D. (2009). Exchange bias in ferrimagnetic–antiferromagnetic nanocomposite produced by mechanical attrition. J. Magn. Magn. Mater..

[21] Fiorani D., Del Bianco L., Testa A.M. (2006). Glassy dynamics in an exchange bias nanogranular system: Fe/FeO*_x_*. J. Magn. Magn. Mater..

[22] Desautels R.D., Skoropata E., Chen Y.-Y., Ouyang H., Freeland J.W., Lierop J.V. (2011). Tuning the surface magnetism of γ-Fe_2_O_3_ nanoparticles with a Cu shell. App. Phys. Lett..

[23] Batlle X., Labarta A. (2002). Finite-size effects in fine particles: Magnetic and transport properties. J. Phys. D.

[24] Ho C.-H., Lai C.-H. (2006). Size-dependent magnetic properties of PtMn nanoparticles. IEEE Trans. Magn..

[25] Dobrynin A.N., Levlev D.N., Temst K., Lievens P., Margueritat J., Gonzalo V., Afonso C.N., Zhou S.Q., Vantomme A., Piscopiello E. (1999). Critical size for exchange bias in ferromagnetic-antiferromagnetic particles. Appl. Phys. Lett..

[26] Sun X., Huls N.F., Sigdel A., Sun S. (2012). Tuning exchange bias in core/shell FeO/Fe_3_O_4_ nanoparticles. Nano Lett..

[27] Dimitriadis V., Kechrakos D., Chubykalo-Fesenko O., Tsiantos V. (2015). Shape-dependent exchange bias effect in magnetic nanoparticles with core-shell morphology. Phys. Rev. B.

[28] Thomas S., Reethu K., Thanveer T., Myint M.T.Z., Al-Harthi S.H. (2017). Effect of shell thickness on the exchange bias blocking temperature and coercivity in Co-CoO core-shell nanoparticles. J. App. Phys..

[29] Cornell R.M., Schwertmann U. (2003). The Iron Oxides: Structure, Properties, Reactions, Occurrences and Uses.

[30] Bellouard C., Mirebeau I., Hennion M. (1996). Magnetic correlations of fine ferromagnetic particles studied by small-angle neutron scattering. Phys. Rev. B.

[31] Phan M.-H., Alonso J., Khurshid H., Lampen-Kelley P., Chandra S., Repa K.S., Nemati Z., Das R., Iglesias O., Srikanth H. (2016). Exchange Bias Effects in Iron Oxide-Based Nanoparticle Systems. Nanomaterials.

[32] Hwang Y., Angappane S., Park J., An K., Hyeon T., Park J.-G. (2012). Exchange bias behavior of monodisperse Fe_3_O_4_/γ-Fe_2_O_3_ core/shell nanoparticles. Curr. Appl. Phys..

[33] Martínez-Boubeta C., Simeonidis K., Angelakeris M., Pazos-Pérez N., Giersig M., Delimitis A., Nalbandian L., Alexandrakis V., Niarchos D. (2006). Critical radius for exchange bias in naturally oxidized Fe nanoparticles. Phys. Rev. B.

[34] Vasilakaki M., Trohidou K.N., Nogués J. (2015). Enhanced Magnetic Properties in Antiferromagnetic-Core/Ferrimagnetic-Shell Nanoparticles. Sci. Rep..

[35] Vasilakaki M., Trohidou K.N. (2009). Numerical study of the exchange-bias effect in nanoparticles with ferromagnetic core/ferrimagnetic disordered shell morphology. Phys. Rev. B.

[36] Häggström L., Kamali S., Ericsson T., Nordblad P., Ahniyaz A., Bergström L. Mössbauer and magnetization studies of iron oxide nanocrystals. Proceedings of the 29th International Conference on the Applications of the Mössbauer Effect (ICAME 2007).

[37] Vandenberghe R.E., de Grave E., Long G.J., Grandjean F. (1989). Mössbauer effect studies of oxidic spinels. Mössbauer Spectroscopy Applied to Inorganic Chemistry.

[38] Lima E., Brandl A.L., Arelaro A.D., Goya G.F. (2006). Spin disorder and magnetic anisotropy in Fe_3_O_4_ nanoparticles. J. Appl. Phys..

[39] Tronc E., Ezzir A., Cherkaoui R., Chanéac C., Noguès M., Kachkachi H., Fiorani D., Testa A.M., Grenèche J.M., Jolivet J.P. (2000). Surface-related properties of γ-Fe_2_O_3_ nanoparticles. J. Mag. Mag. Mater..

[40] Brice-Profeta S., Arrio M.-A., Tronc E., Menguy N., Letard I., Cartier dit Moulin C., Noguès M., Chanéac C., Jolivet J.-P., Sainctavit P. (2005). Magnetic order in γ-Fe_2_O_3_ nanoparticles: A XMCD study. J. Mag. Mag. Mater..

[41] Gabbasov R.R., Cherepanov V.M., Chuev M.A., Polikarpov M.A., Panchenko V.Y. (2014). Size effect of Mossbauer parameters in iron oxide Nanoparticles. Hyperfine Interact..

[42] Siddique M., Hussain N., Shafi M. (2009). Identification of Iron Oxides Qualitatively/Quantitatively Formed during the High Temperature Oxidation of Superalloys in Air and Steam Environments. J. Mater. Sci. Technol..

[43] Rui W.B., Hu Y., Du A., You1 B., Xiao M.W., Zhang W., Zhou S.M., Du J. (2015). Cooling field and temperature dependent exchange bias in spin glass/ferromagnet bilayers. Sci. Rep..

[44] Rizwan Ali S., Bilal Janjua M., Fecioru-Morariu M., Lott D., Smits C.J.P., Güntherodt G. (2010). Role of interface alloying in the exchange bias of Fe/Cr bilayers. Phys. Rev. B.

[45] Ali M., Adie P., Marrows C.H., Greig D., Hickey B.J., Stamps R.L. (2007). Exchange bias using a spin glass. Nat. Mater..

[46] Nolle D., Goering E., Tietze T., Schütz G., Figuerola A., Manna L. (2009). Structural and magnetic deconvolution of FePt/FeO*_x_*-nanoparticles using X-ray magnetic circular dichroism. New J. Phys..

[47] Tan A., Li J., Jenkins C.A., Arenholz E., Scholl A., Hwang C., Qiu Z.Q. (2012). Exchange bias in epitaxially grown CoO/MgO/Fe/Ag(001). Phys. Rev. B..

[48] Zhou S.M., Imhoff D., Yu-Zhang K., Leprince-Wang Y. (2015). Effect of field cooling on magnetic properties of ultrafine CoO/Co particles. Appl. Phys. A Mater. Sci. Process.

[49] Luna C., Ilyn M., Vega V., Prida V.M., González J., Mendoza-Reśendez R. (2014). Size Distribution and Frustrated Antiferromagnetic Coupling Effects on the Magnetic Behavior of Ultrafine Akaganéite (β-FeOOH) Nanoparticles. J. Phys. Chem. C.

[50] Tang Y.-K., Sun Y., Cheng Z.-H. (2006). Exchange bias associated with phase separation in the perovskite cobaltite La_1−*x*_Sr*_x_*CoO_3_. Phys. Rev. B.

[51] Grosvenor A.P., Kobe B.A., McIntyre N.S. (2004). Examination of the oxidation of iron by oxygen using X-ray photoelectron spectroscopy and QUASES TM. Surf. Sci..

